# Supplementary material for: Extensive Mendelian randomization study identifies potential causal risk factors for severe COVID-19
Source: Commun Med (Lond). 2021 Dec 9;1:59. doi: 10.1038/s43856-021-00061-9 (PMC9053245; doi:10.1038/s43856-021-00061-9)
Supplement: Supplementary file 16 — Description of Additional Supplementary Files [file 43856_2021_61_MOESM16_ESM.pdf]

Description of Additional Supplementary Files

File Name: Supplementary Data 1

Description: Existing Mendelian randomization studies of COVID-19.

File Name: Supplementary Data 2

Description: STROBE-MR checklist.

File Name: Supplementary Data 3

Description: Details of the datasets, traits and GWAS used in the present Mendelian randomization study.

nsnp, number of SNPs; ncase, number of cases; ncontrol, number of controls; sd, standard deviation; NA, not available.

File Name: Supplementary Data 4

Description: Significant and twice-replicated causal associations with severe COVID-19 (IVW FDR < 0.05 with release 4 alpha HGI A2; and IVW $p < 0.05$ with release 4 alpha HGI B2 and IVW $p < 0.05$ with NEJM).

Only associations that are replicated in both replication analyses (with the HGI B2 and NEJM datasets) are included here. Odds ratios and 95% confidence intervals were derived using the inverse-variance weighted random-effects model. The full list, including those that are replicated with only one study, is available in Supplementary Data 5. SNP, single nucleotide polymorphism; SNP#, number of SNPs retained for this analysis; CI, confidence interval.

File Name: Supplementary Data 5

Description: Significant and replicated results (IVW FDR < 0.05 with release 4 alpha HGI A2; and IVW $p < 0.05$ with release 4 alpha HGI B2 OR IVW $p < 0.05$ with NEJM).

Note for "Type of findings": Novel, not reported before; Confirming, confirming some previously reported results, although previous results may be conflicting among themselves; Conflicting, conflicting with previously reported results. b: causal effect size; se: standard error; pval: p-

value; IVW_MRE: inverse-variance weighted random-effects model; Egger: MR-Egger; Wald: Wald ratio; Het: heterogeneity; W_Med: weighted median; W_Mod: weighted mode; nsnps: number of SNPs retained for this analysis; qval: q-value.

File Name: Supplementary Data 6

Description: Suggestive and replicated results (IVW $p < 0.05$ with release 4 alpha HGI A2; and IVW $p < 0.05$ with release 4 alpha HGI B2 OR IVW $p < 0.05$ with NEJM).

Note for "Type of findings": Novel, not reported before; Confirming, confirming some previously reported results, although previous results may be conflicting among themselves; Conflicting, conflicting with previously reported results. b: causal effect size; se: standard error; pval: p-value; IVW_MRE: inverse-variance weighted random-effects model; Egger: MR-Egger; Wald: Wald ratio; Het: heterogeneity; W_Med: weighted median; W_Mod: weighted mode; nsnps: number of SNPs retained for this analysis; qval: q-value.

File Name: Supplementary Data 7

Description: Supplementary Data 7
Description: All MR results based on release 4 alpha HGI A2 and B2, and NEJM.

b: causal effect size; se: standard error; pval: p-value; IVW_MRE: inverse-variance weighted random-effects model; Egger: MR-Egger; Wald: Wald ratio; Het: heterogeneity; W_Med: weighted median; W_Mod: weighted mode; nsnps: number of SNPs retained for this analysis; qval: q-value.

File Name: Supplementary Data 8

Description: Multivariable Mendelian randomization of fat mass and fat-free mass indices on severe COVID-19, based on the release 4 alpha.

Beta: causal effect size; SE: standard error; pval: p-value; MVMR: multivariable Mendelian randomization.

File Name: Supplementary Data 9

Description: All MR results based on release 4 HGI A2 and B2, and release 5 HGI A2 and B2.

b: causal effect size; se: standard error; pval: p-value; IVW_MRE: inverse-variance weighted random-effects model; Egger: MR-Egger; Wald: Wald ratio; Het: heterogeneity; W_Med: weighted median; W_Mod: weighted mode; nsnps: number of SNPs retained for this analysis.

File Name: Supplementary Data 10

Description: All MR results based on release 4 HGI A1 and B1, and release 5 HGI B1.

b: causal effect size; se: standard error; pval: p-value; IVW_MRE: inverse-variance weighted random-effects model; Egger: MR-Egger; Wald: Wald ratio; Het: heterogeneity; W_Med: weighted median; W_Mod: weighted mode; nsnps: number of SNPs retained for this analysis.

File Name: Supplementary Data 11

Description: Genetic correlation of COVID-19 GWAS across phenotype definitions and data releases.

Note: Because the mean chi-square was below 1.02, we could not apply LDSC regression to assess the genetic correlations of release 4 (A1, B1, and C1) and 5 (B1and B1 leaving out UK Biobank). Some of the genetic correlation estimates were out of bounds (e.g., $r_g > 1$), because the heritability estimates (h^2) were very low for some COVID-19 phenotypes. r_g: genetic correlation estimates; se: standard error; pval: p-value; h2_obs: total observed heritability of phenotype 1; int: intercept; gcov: genetic covariance; R4: release 4; R5: release 5; NO_UKB: leaving out UK Biobank.

File Name: Supplementary Data 12

Description: All MR results based on release 4 HGI C2, and release 5 HGI C2 and C2 leaving out UK Biobank.

b: causal effect size; se: standard error; pval: p-value; IVW_MRE: inverse-variance weighted random-effects model; Egger: MR-Egger; Wald: Wald ratio; Het: heterogeneity; W_Med: weighted median; W_Mod: weighted mode; nsnps: number of SNPs retained for this analysis.

File Name: Supplementary Data 13

Description: Comparisons of severity risk factors with susceptibility risk factors. There are three groups. First, the shared risk factors: significant and replicated severity risk factors that overlap with susceptibility risk factors based on HGI C2 dataset (IVW $p < 0.05$ with release 4 HGI C2, release 5 HGI C2, or release 5 HGI C2 leaving out UK Biobank). Second, the severity-only risk factors: significant and replicated severity risk factors that do not overlap with susceptibility risk factors based on HGI C2 dataset (IVW $p > 0.05$ with release 4 HGI C2, release 5 HGI C2, and release 5 HGI C2 leaving out UK Biobank). Third, the susceptibility-only risk factors: suggestive susceptibility risk factors based on the C2 datasets (IVW $p < 0.05$ with release 4 HGI C2,

release 5 HGI C2, and release 5 HGI C2 leaving out UK Biobank; but not in release 4 alpha HGI A2 or HGI B2 or NEJM).

b: causal effect size; se: standard error; pval: p-value; IVW_MRE: inverse-variance weighted random-effects model; Egger: MR-Egger; Wald: Wald ratio; Het: heterogeneity; W_Med: weighted median; W_Mod: weighted mode; nsnps: number of SNPs retained for this analysis; A2: release 4 alpha A2; B2: release 4 alpha B2; R4_C2: release 4 C2; R5_C2: release 5 C2; R5_C2_NO_UKB: release 5 C2, leaving out UK Biobank. NA indicates that there are no reliable estimates, mostly due to pleiotropy in genetic instruments.

File Name: Supplementary Data 14

Description: Known associations of circulating proteins-associated and glucosamine-associated SNPs in PhenoScanner at genome-wide significance.

Pos: position; EA: effect allele; NEA:non effect allele; b: causal effect size; se: standard error; pval: p-value; SNPs were identified by searching the PhenoScanner database (http://www.phenoscanner.medschl.cam.ac.uk/).

File Name: Supplementary Data 15

Description: Sensitivity analysis of circulating proteins and glucosamine supplements after excluding SNPs with potential pleiotropy.

b: causal effect size; se: standard error; pval: p-value; IVW_MRE: inverse-variance weighted random-effects model; Egger: MR-Egger; Wald: Wald ratio; Het: heterogeneity; W_Med: weighted median; W_Mod: weighted mode; nsnps: number of SNPs retained for this analysis.
