# Supplementary material for: Extensive Mendelian randomization study identifies potential causal risk factors for severe COVID-19
Source: Commun Med (Lond). 2021 Dec 9;1:59. doi: 10.1038/s43856-021-00061-9 (PMC9053245; doi:10.1038/s43856-021-00061-9)
Supplement: Supplementary file 18 — Supplementary Information [file 43856_2021_61_MOESM18_ESM.pdf]

Supplementary Information for

Extensive Mendelian randomization study identifies potential causal risk factors for severe COVID-19

Yitang Sun[1], Jingqi Zhou[1,2], Kaixiong Ye[1,3,*]

[1]Department of Genetics, Franklin College of Arts and Sciences, University of Georgia, Athens, GA, USA. [2]School of Public Health, Shanghai Jiao Tong University School of Medicine, Shanghai, China. [3]Institute of Bioinformatics, University of Georgia, Athens, GA, USA.

*** Address for Correspondence:**

Dr. Kaixiong Ye
Department of Genetics
University of Georgia
C220 Davison Life Sciences
120 East Green Street, Athens, GA 30602
Office: 706-542-5898
Fax: 706-542-3910
Email: Kaixiong.Ye@uga.edu

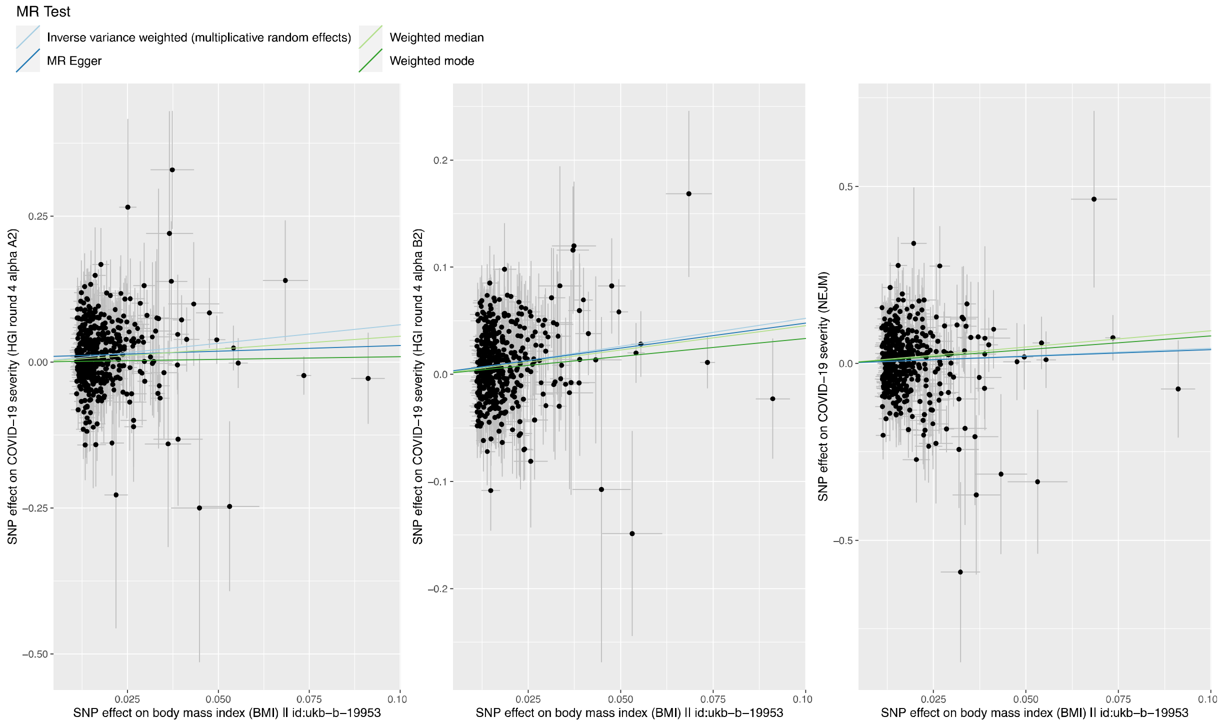

Supplementary Figure 1. Scatter plot of SNP-BMI associations versus SNP-COVID-19 severity associations for MR analyses. Genetic association estimates of BMI instrumental variables were not detected to be outliers in A2, B2, and NEJM studies. Horizontal error bars represent standard errors of BMI estimates, while vertical error bars represent standard errors of COVID-19 severity estimates. The light blue line represents the inverse-variance weighted random-effects causal estimate of BMI on COVID-19 severity. The dark blue line represents the MR-Egger causal estimate of BMI on COVID-19 severity. The light green line represents the weighted median causal estimate of BMI on COVID-19 severity. The dark green line represents the weighted mode causal estimate of BMI on COVID-19 severity.

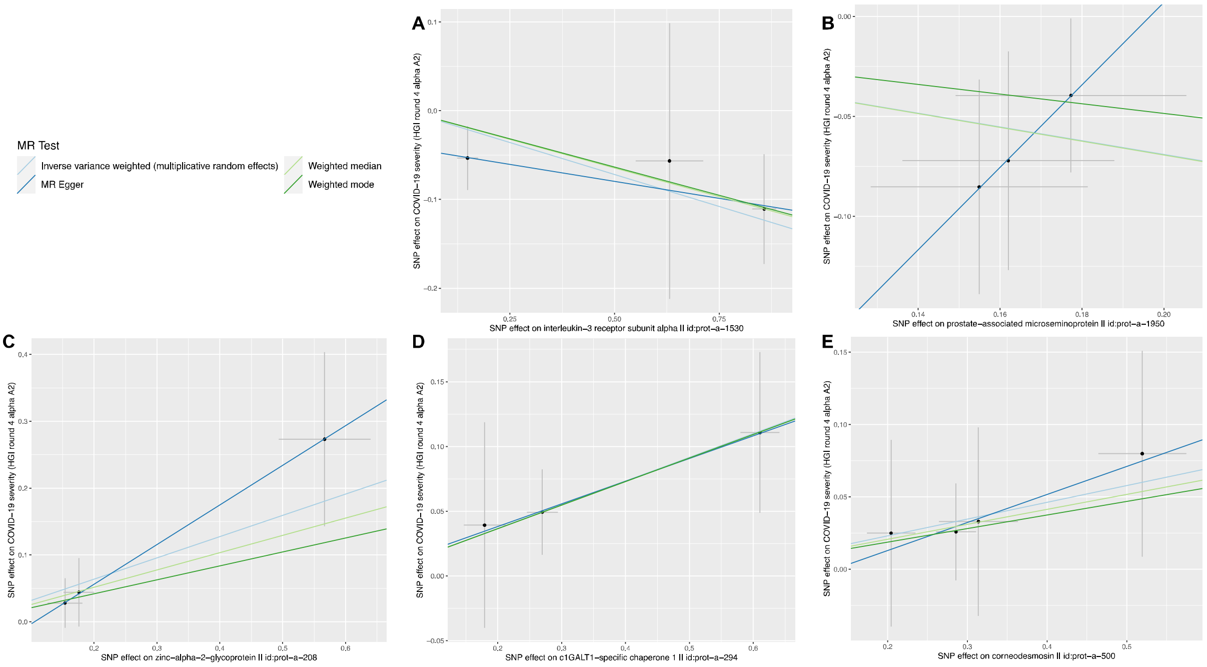

Supplementary Figure 2. Scatter plot of SNP-circulating proteins associations versus SNP-COVID-19 severity associations for MR analyses based on the release 4 alpha HGI A2 dataset. (A) interleukin-3 receptor subunit alpha, (B) prostate-associated microseminoprotein, (C) zinc-alpha-2-glycoprotein, (D) C1GALT1-specific chaperone 1, and (E) corneodesmosin. Horizontal error bars represent standard errors of exposure estimates, while vertical error bars represent standard errors of COVID-19 severity estimates. The light blue line represents the inverse-variance weighted random-effects causal effect of proteins on COVID-19 severity. The dark blue line represents the MR-Egger causal effect of proteins on COVID-19 severity. The light green line represents the weighted median causal effect of proteins on COVID-19 severity. The dark green line represents the weighted mode causal effect of proteins on COVID-19 severity.

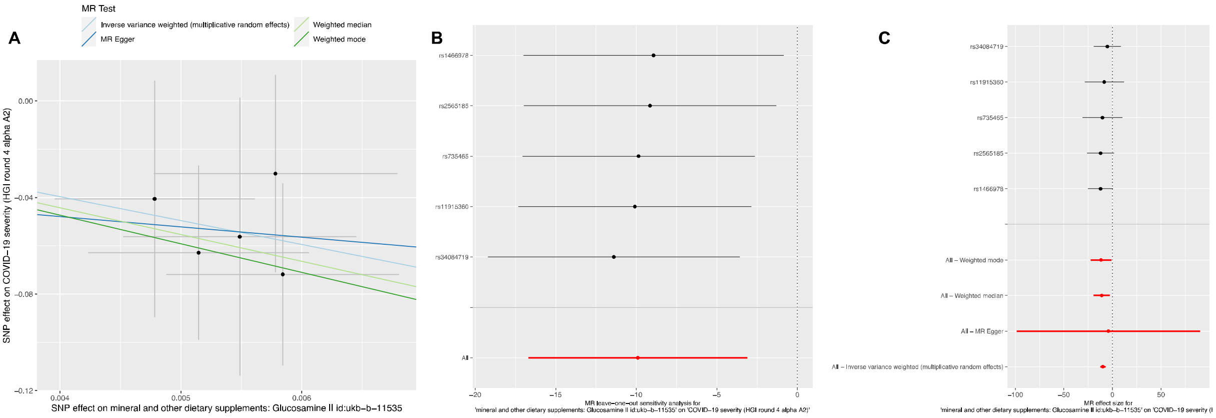

Supplementary Figure 3. The causal effect of glucosamine supplement on COVID-19 severity based on the release 4 alpha HGI A2 dataset. (A) Scatter plot for glucosamine supplement and COVID-19 severity. Horizontal error bars represent standard errors of glucosamine supplement estimates, while vertical error bars represent standard errors of COVID-19 severity estimates. (B) Leave-one-out analysis for glucosamine supplement and COVID-19 severity. (C) A forest plot for the causal effect of glucosamine supplement on COVID-19 severity with different SNPs and MR tests. Bars represent 95% confidence intervals.

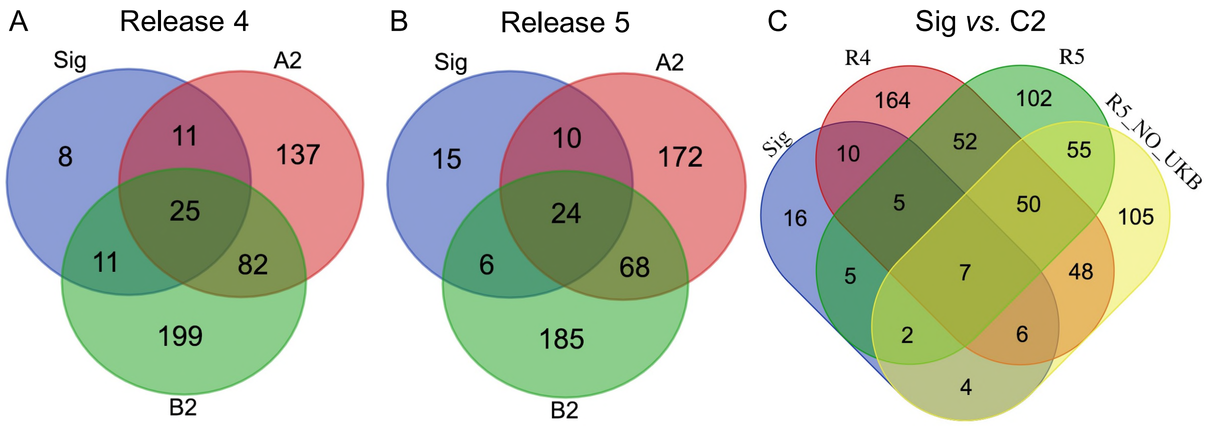

Supplementary Figure 4. Venn diagrams for comparisons of significant and replicated results with candidate exposures based on various HGI GWAS and releases. (A) Comparisons with candidate exposures based on COVID-19 GWAS (A2 and B2) from HGI release 4. (B) Comparisons with candidate exposures based on COVID-19 GWAS (A2 and B2) from HGI release 5. (C) Comparisons with candidate exposures based on HGI C2 GWAS from different releases. Sig, significant and replicated results from our primary analysis based on HGI release 4 alpha and NEJM (IVW FDR < 0.05 with release 4 alpha HGI A2; and IVW p < 0.05 with release 4 alpha HGI B2 OR IVW p < 0.05 with NEJM); R4, HGI release 4; R5, HGI release 5; R5_NO_UKB, HGI release 5 leaving out UK Biobank.
