# Supplementary material for: Extensive Mendelian randomization study identifies potential causal risk factors for severe COVID-19
Source: Commun Med (Lond). 2021 Dec 9;1:59. doi: 10.1038/s43856-021-00061-9 (PMC9053245; doi:10.1038/s43856-021-00061-9)
Supplement: Supplementary file 19 — Reporting Summary [file 43856_2021_61_MOESM19_ESM.pdf]

nature research

Corresponding author(s): K Ye

Last updated by author(s): Nov 16, 2021

Reporting Summary

Nature Research wishes to improve the reproducibility of the work that we publish. This form provides structure for consistency and transparency in reporting. For further information on Nature Research policies, see our Editorial Policies and the Editorial Policy Checklist.

Statistics

For all statistical analyses, confirm that the following items are present in the figure legend, table legend, main text, or Methods section.

n/a	Confirmed	
☐	☒	The exact sample size (*n*) for each experimental group/condition, given as a discrete number and unit of measurement
☒	☐	A statement on whether measurements were taken from distinct samples or whether the same sample was measured repeatedly
☒	☐	The statistical test(s) used AND whether they are one- or two-sided<br>*Only common tests should be described solely by name; describe more complex techniques in the Methods section.*
☒	☐	A description of all covariates tested
☐	☒	A description of any assumptions or corrections, such as tests of normality and adjustment for multiple comparisons
☐	☒	A full description of the statistical parameters including central tendency (e.g. means) or other basic estimates (e.g. regression coefficient) AND variation (e.g. standard deviation) or associated estimates of uncertainty (e.g. confidence intervals)
☐	☒	For null hypothesis testing, the test statistic (e.g. *F*, *t*, *r*) with confidence intervals, effect sizes, degrees of freedom and *P* value noted<br>*Give P values as exact values whenever suitable.*
☒	☐	For Bayesian analysis, information on the choice of priors and Markov chain Monte Carlo settings
☒	☐	For hierarchical and complex designs, identification of the appropriate level for tests and full reporting of outcomes
☐	☒	Estimates of effect sizes (e.g. Cohen's *d*, Pearson's *r*), indicating how they were calculated

Our web collection on statistics for biologists contains articles on many of the points above.

Software and code

Policy information about availability of computer code

Data collection	The R package TwoSampleMR (version 0.5.5) was applied to retrieve the IEU GWAS datasets.
Data analysis	The codes used in the Mendelian randomization analyses described here are freely accessible in TwoSampleMR R package via GitHub (https://github.com/MRCIEU/TwoSampleMR/). Full documentation for the R package is also provided (https://mrcieu.github.io/TwoSampleMR/). We implemented the MVMR analysis using the MVMR R package (https://github.com/WSpiller/MVMR/). The Mendelian randomization pipeline is available on GitHub (https://github.com/yitangsun/MR_all_COVID_19) and also deposited on Zenodo83.

For manuscripts utilizing custom algorithms or software that are central to the research but not yet described in published literature, software must be made available to editors and reviewers. We strongly encourage code deposition in a community repository (e.g. GitHub). See the Nature Research guidelines for submitting code & software for further information.

Data

Policy information about availability of data

All manuscripts must include a data availability statement. This statement should provide the following information, where applicable:

- Accession codes, unique identifiers, or web links for publicly available datasets
- A list of figures that have associated raw data
- A description of any restrictions on data availability

Source data for the Figures 2 and 3 could be found in Supplementary Data 8, and that for Figure 4 in Supplementary Data 6. All analyses were conducted using publicly available data. The exposure data (GWAS summary statistics) used in the analyses described here are freely accessible in the MR-Base platform (https://www.mrbase.org/) and the IEU OpenGWAS database (https://gwas.mrcieu.ac.uk/). Unique identifier for each dataset is available in Supplementary Data 3. We downloaded COVID-19 data (GWAS summary statistics) in the COVID-19 Host Genetics Initiative (https://www.covid19hg.org/) and the COVID GWAS results browser (https://ikmb.shinyapps.io/COVID-19_GWAS_Browser/).

April 2020

Field-specific reporting

Please select the one below that is the best fit for your research. If you are not sure, read the appropriate sections before making your selection.

☒ Life sciences ☐ Behavioural & social sciences ☐ Ecological, evolutionary & environmental sciences

For a reference copy of the document with all sections, see nature.com/documents/nr-reporting-summary-flat.pdf

Life sciences study design

All studies must disclose on these points even when the disclosure is negative.

Sample size	We did not perform any sample size calculations. We used existing GWAS summary statistics.
Data exclusions	Exposure GWAS were filtered based on the following criteria: 1) European ancestry; 2) not eQTL studies, those labeled as “eqtl” from eQTLGen 2019. A total of 14,385 GWAS summary datasets were retained and used in this study. Two additional exclusion criteria were applied to filter out exposures before FDR correction: 1) the number of genetic instruments was less than three. Three or more are required for statistical tests of pleiotropic effects and for statistical sensitivity analyses to correct for pleiotropy. 2) Exposures with indications of pleiotropy in their genetic instruments. The presence of pleiotropy violates the assumption of MR analysis.
Replication	Results were replicated using different GWAS of COVID-19 from HGI, although these GWAS are not independent of each other.
Randomization	Mendelian randomization uses randomly allocated genetic variants to obtain unconfounded estimates (following Mendel's laws of inheritance).
Blinding	This is not relevant to our study.

Reporting for specific materials, systems and methods

We require information from authors about some types of materials, experimental systems and methods used in many studies. Here, indicate whether each material, system or method listed is relevant to your study. If you are not sure if a list item applies to your research, read the appropriate section before selecting a response.

Materials & experimental systems

n/a	Involved in the study	
☒	☐	Antibodies
☒	☐	Eukaryotic cell lines
☒	☐	Palaeontology and archaeology
☒	☐	Animals and other organisms
☒	☐	Human research participants
☒	☐	Clinical data
☒	☐	Dual use research of concern

Methods

n/a	Involved in the study	
☒	☐	ChIP-seq
☒	☐	Flow cytometry
☒	☐	MRI-based neuroimaging
